# Molecular detection of enteric viruses and the genetic characterization of porcine astroviruses and sapoviruses in domestic pigs from Slovakian farms

**DOI:** 10.1186/s12917-018-1640-8

**Published:** 2018-10-19

**Authors:** Slavomira Salamunova, Anna Jackova, Rene Mandelik, Jaroslav Novotny, Michaela Vlasakova, Stefan Vilcek

**Affiliations:** University of Veterinary Medicine and Pharmacy, Komenskeho 73, 040 00 Kosice, Slovakia

**Keywords:** Porcine astrovirus, Porcine sapovirus, Diarrhea, Pig, Phylogenetic analysis

## Abstract

**Background:**

Surveillance and characterization of pig enteric viruses such as transmissible gastroenteritis virus (TGEV), porcine epidemic diarrhea virus (PEDV), rotavirus, astrovirus (PAstV), sapovirus (PSaV), kobuvirus and other agents is essential to evaluate the risks to animal health and determination of economic impacts on pig farming. This study reports the detection and genetic characterization of PAstV, PSaV in healthy and diarrheic domestic pigs and PEDV and TGEV in diarrheic pigs of different age groups.

**Results:**

The presence of PAstV and PSaV was studied in 411 rectal swabs collected from healthy (*n* = 251) and diarrheic (*n* = 160) pigs of different age categories: suckling (*n* = 143), weaned (*n* = 147) and fattening (*n* = 121) animals on farms in Slovakia. The presence of TGEV and PEDV was investigated in the diarrheic pigs (*n* = 160). A high presence of PAstV infections was detected in both healthy (94.4%) and diarrheic (91.3%) pigs. PSaV was detected less often, but also equally in clinically healthy (8.4%) and diarrheic (10%) pigs. Neither TGEV nor PEDV was detected in any diarrheic sample. The phylogenetic analysis of a part of the RdRp region revealed the presence of all five lineages of PAstV in Slovakia (PAstV-1 – PAstV-5), with the most frequent lineages being PAstV-2 and PAstV-4. Analysis of partial capsid genome sequences of the PSaVs indicated that virus strains belonged to genogroup GIII. Most of the PSaV sequences from Slovakia clustered with sequences originating from neighbouring countries.

**Conclusions:**

Due to no significant difference between healthy and diarrheic pigs testing of the presence of PAstV and PSaV provides no diagnostic value. Genetic diversity of PAstV was very high as all five lineages were identified in pig farms in Slovakia. PSaV strains were genetically related to the strains circulating in Central European region.

## Background

Viral gastroenteritis is one of the serious diseases in pigs with high morbidity observed worldwide, causing significant financial losses. Therefore, surveillance and characterization of pig enteric viruses such as transmissible gastroenteritis virus (TGEV), porcine epidemic diarrhea virus (PEDV), rotavirus, astrovirus, sapovirus, kobuvirus and other agents is essential to evaluate the possible risks to animal health as well as for the epidemiological analysis and determination of economic impacts on pig farming.

Many enteric RNA viruses have been found in swine herds including those from genera *Mamastrovirus* and *Sapovirus.* The astroviruses from genus *Mamastrovirus,* family *Astroviridae* affect a wide variety of mammalian species [1]. They consist of small (28–30 nm), non-enveloped, single-stranded, positive-sense RNA molecule of 6.4–7.7 kb in size. Their genome is arranged in three open reading frames (ORF). The ORF1a and ORF1b at the 5’end encode non-structural proteins and ORF2 at the 3′ end encodes the structural proteins [1].

Porcine astrovirus (PAstV) isolated from pigs worldwide [2–4] includes five highly genetic variable lineages (PAstV-1 to PAstV-5) [4, 5]. In Europe, all five lineages have been detected [6], with the most common lineage being PAstV-4. PAstV has also been detected in several Central European countries [7, 8].

Genus *Sapovirus* belongs within the family *Caliciviridae.* Similar to astrovirus, it is a non-enveloped virus, containing a single-stranded, positive-sense RNA genome of 7.4–8.3 kb. The virus genome is organized into two or three ORFs, which encode both structural and non-structural proteins, including the RNA-dependent RNA polymerase (RdRp) and the major capsid protein (VP1) [1]. Due to great genetic variability and recombination capacity, the genus is divided into fifteen different genogroups based on analysis of both capsid and RdRp sequences [9, 10]. Sapoviruses infect a wide variety of hosts including humans, pigs, mink, canines and bats [11–14]. The porcine sapovirus (PSaV), formerly known as porcine enteric calicivirus (PEC), is divided into GIII, GV – GXI genogroups, but GIII is considered as the prototypic genogroup with the only cultivable Cowden strain [9].

While representatives of the genera *Mamastrovirus* and *Sapovirus* were mainly associated with gastroenteritis in infants and young children [1, 15], the epidemiology of these viruses in animals especially in pigs is not so well understood and is intensively studied. The virus infections are associated with enteric diseases, but their role in diarrhea or gastroenteritis is still unknown due their detection in healthy as well as in diarrheic pigs [7, 16].

TGEV and PEDV, however, are considered to be important causative agents of diarrhea in pigs. TGEV and PEDV are members of the genus *Alphacoronavirus* of the family C*oronaviridae* with enveloped virions, containing longer positive single-stranded RNA genomes approximately 28 kb in length [1]. These viruses are causative agents of acute diarrhea in all age categories and can cause high mortality in neonatal piglets, resulting in significant economic losses [17].

In Slovakia, there is limited information on the occurrence of enteric viruses in pig farms so far. We have selected for our research project three important enteric viruses, namely rotavirus A (RVA), TGEV and PEDV. In addition we were also interested in porcine kobuvirus 1 – PKV-1, PAstV and PSaV, which are believed to be enteric viruses too. Recently we have analysed a broad collection of pig rectal swabs for the detection of RVA and PKV-1 [18]. This study on the same samples reports the molecular detection and partial genetic characterization of PAstV, PSaV in healthy and diarrheic domestic pigs and PEDV and TGEV in diarrheic domestic pigs of different age groups.

## Results

### Molecular detection of TGEV and PEDV

Neither TGEV nor PEDV was detected in any of the rectal swabs collected from 160 diarrheic pigs.

### Molecular detection of porcine astrovirus

**Fig. 1:**
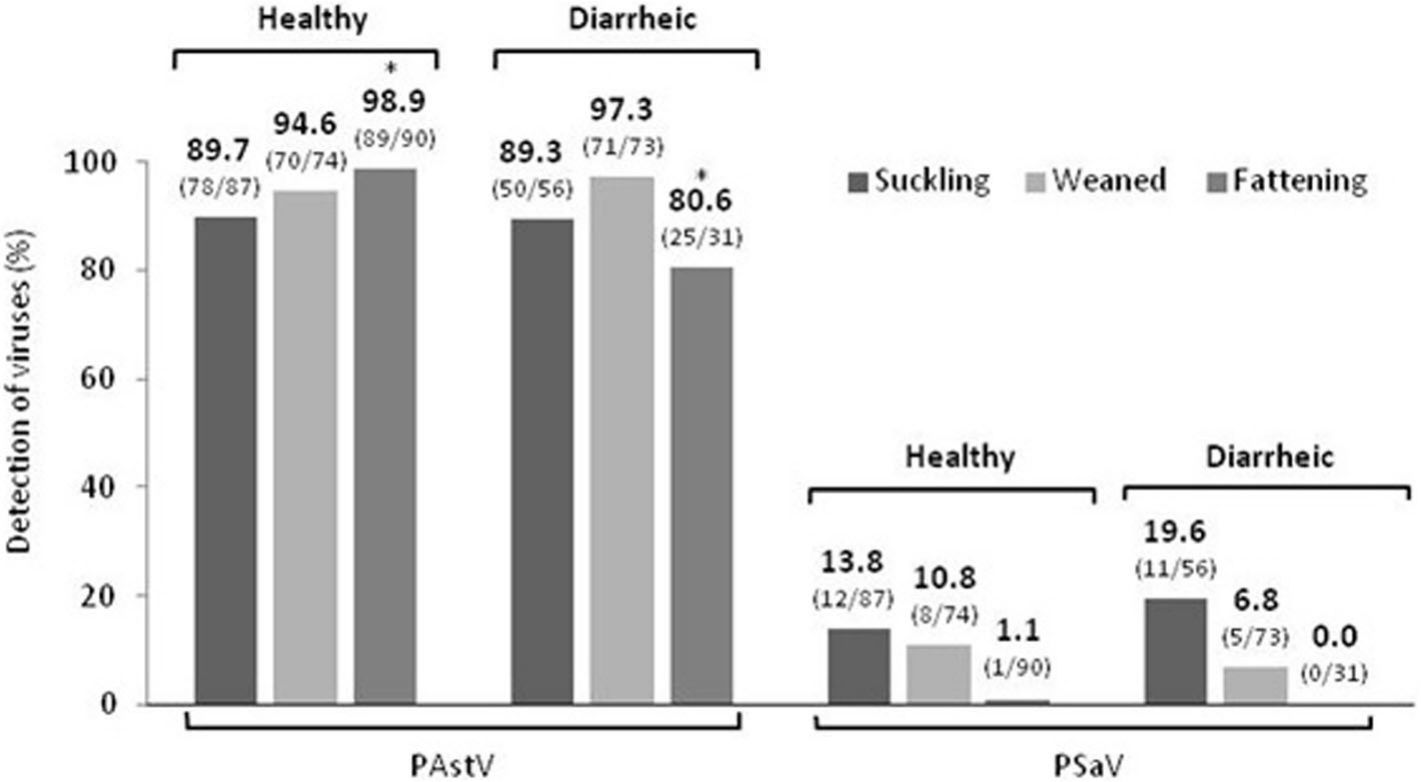
PAstV and PSaV in healthy and diarrheic pigs of three age categories. Brackets indicate positive/total number of samples analysed. The star indicates statistically significant (*p* < 0.01) value

Results of the PAstV detection in investigated pigs are presented on Fig. 1.

PAstV RNA was detected in all 17 investigated farms with an overall prevalence 93.2% (383/411).

Among healthy pigs 94.4% (237/251) were found to be infected by PAstV. The most affected group across age categories was the fattening pigs where nearly all animals were virus positive (98.9%; 89/90) followed by weaned pigs (94.6%; 70/74), and suckling piglets (89.7%; 78/87).

In diarrheic animals, 91.3% (146/160) pigs were infected by PAstV. The most affected group across age categories were weaned pigs (97.3%; 71/73), followed by suckling piglets (89.3%; 50/56) and fattening pigs (80.6%; 25/31).

Statistical analysis of individual age categories revealed high significant differences of astrovirus infection between diarrheic fattening (80.6%) pigs and healthy fattening (98.6%) pigs (*p* < 0.01, χ^2^ = 14.08) with even higher prevalence in healthy animals. No significant differences were observed between healthy and diarrheic in the other two age categories.

### Molecular detection of porcine sapovirus

The values of detection of PSaV in pigs are presented on Fig. 1.

Only 7 of the 17 farms investigated were positive for PSaV RNA. Of all investigated samples, 9% (37/411) were virus positive.

Approximately the same number of animals were infected by virus in the group of healthy pigs (8.4% - 21/251) as in diarrheic (10% - 16/160) animals. In both, healthy and diarrheic animals, the most affected group across age categories were suckling piglets (13.8% - 12/87 healthy and 19.6% - 11/56 diarrheic pigs), followed by weaned pigs (10.8% - 8/74 and 6.8% - 5/73). None of the diarrheic pigs and only one positive sample (1.1% - 1/90) of PSaV in healthy pigs was detected in the fattening age category. All PSaV positive pigs were also infected with PAstV.

There were no statistically significant differences between healthy and diarrheic groups of pigs in any of the three age categories mentioned above.

### Sequence and phylogenetic analysis of partial RdRp region of PAstV

**Fig. 2:**
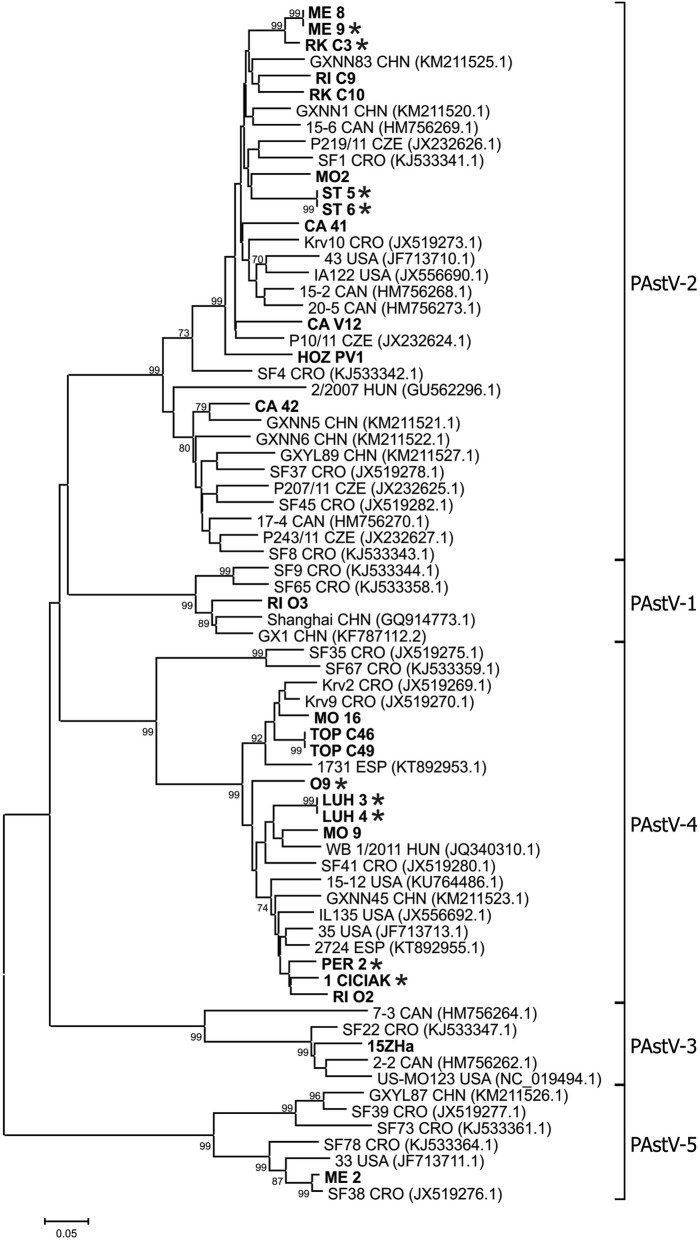
Phylogenetic tree of PAstV. The tree was constructed on the base of 355 to 382 bp (depend on PAstV lineage) nucleotide sequences of RdRp of the PAstV and selected sequences from GenBank (labelled with accession number and country of origin). Sequences from Slovakian samples are in bolt, from diarrheic pigs are marked with stars. The tree was constructed using the neighbour-joining method implemented in MEGA6 software. Scale bar represents the number of nucleotide substitutions per site

Twenty five amplicons from PAstV positive samples coming from 13 farms were selected for nucleotide sequencing. The phylogenetic analysis of RdRp region fragments is shown in Fig. 2.

Five lineages PAstV-1 to PAstV-5 were detected in investigated pigs from Slovakia. The lineages PAstV-2 (*n* = 12) and PAstV-4 (*n* = 10) were the most represented. The remaining tree lineages were each represented by one sequence. The nucleotide sequence identities between Slovakian PAstV lineages were 52.0–65.4%. Variability of Slovakian sequences inside the lineage PAstV-2 was in the range 74.9–100%, and variability inside the lineage PAstV-4 was 84.6–100%.

All sequences from Slovakian samples clustered together with viral sequences from European countries, particularly Croatia, but also with sequences from North America and China. The clustering of Slovakian sequences with those of neighbouring countries was sporadic. Farm-specific grouping was observed with the most of PAstV sequences. However, isolates circulating on one farm belonging to 2–3 different lineages were confirmed on three investigated farms (Fig. 2, farms ME, MO and RI).

### Sequence and phylogenetic analysis of partial capsid protein of PSaV

**Fig. 3:**
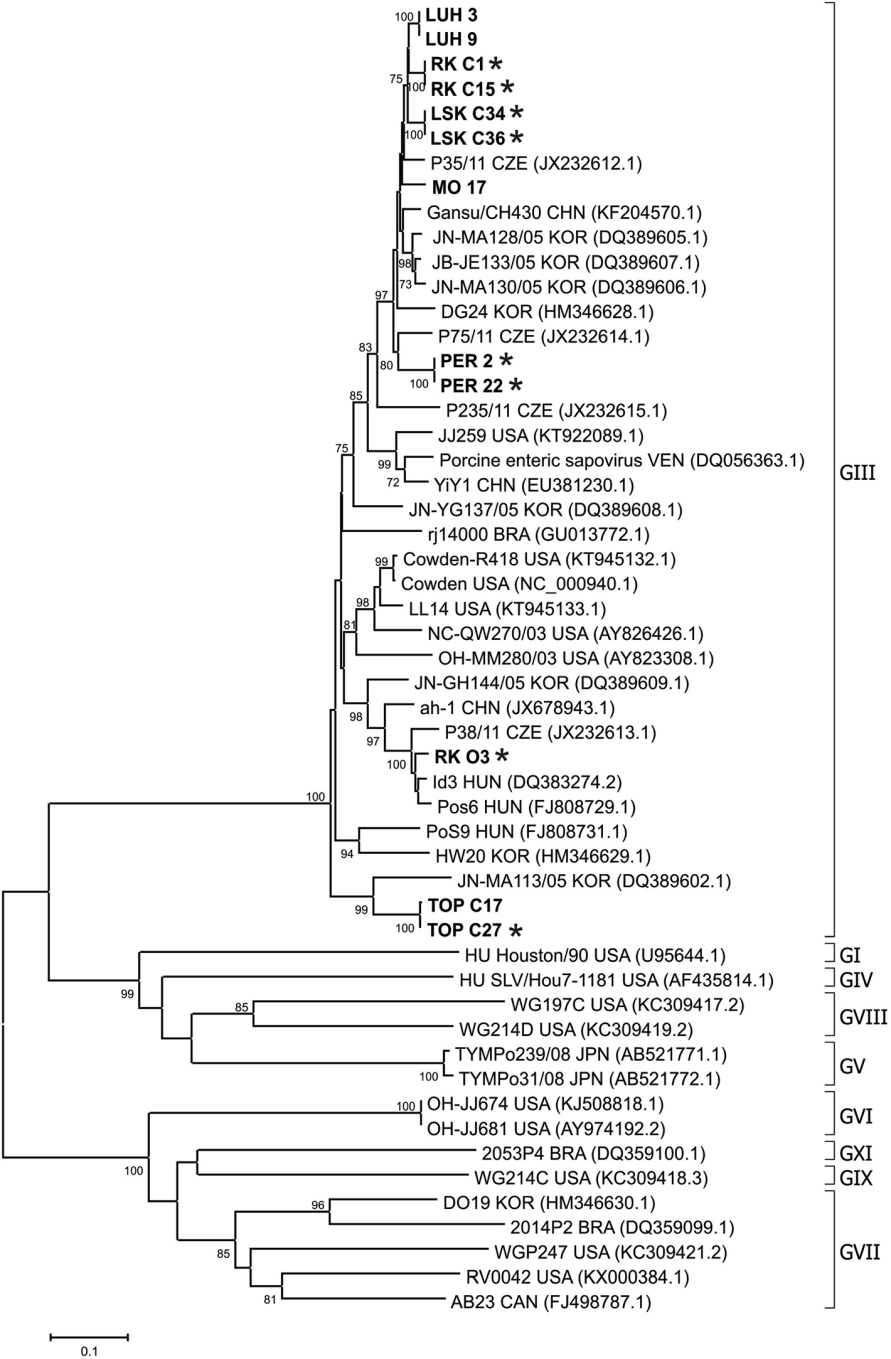
Phylogenetic tree of PSaV. The tree was constructed on the base of 667 bp nucleotide sequences of capsid PSaV and selected sequences from GenBank (labelled with accession number and country of origin). Two references human strains from GenBank are also included (HU). Sequences from Slovakian samples are in bolt, from diarrheic pigs are marked with stars. The tree was constructed using the neighbour-joining method implemented in MEGA6 software. Scale bar represents the number of nucleotide substitutions per site

Twelve PSaV PCR amplicons were sequenced, which were selected to include the majority of infected farms, different health status and age categories of pigs. The phylogenetic analysis of 667 bp fragment (capsid region) is shown in Fig. 3.

All of the investigated viral sequences belong to genogroup GIII, showing nucleotide sequence identities in the range 78.7–100%. On the phylogenetic tree, all sequences from Slovakian pig samples clustered together with viral sequences from neighbouring countries, such as Czech Republic and Hungary, except two strains TOP C17 and TOP C27 which were clustered together with a sequence from Korea. PSaV sequences were clustered according to the farm origin, except RK O3 which was separate from sequences originating from the same farm.

## Discussion

In this work, we have determined for the first time the presence of the PAstV and PSaV infections in pigs from Slovakia. PAstV has been detected widely in rectal swab samples of healthy and diarrheic pigs of all age categories as demonstrated by a greater than 90% prevalence of the virus. On the other hand, the incidence of PSaV has been found to be more sporadic (8–10%) in pig samples investigated independently if they originated from healthy or diarrheic pigs. Our results indicate that there is no strong correlation between detection of both viruses in enteric samples and diarrhea. This observation has been quite surprising as it is believed that porcine astrovirus and porcine sapovirus belong to the common agents of pig gastrointestinal infections, which are usually associated with diarrheic diseases [15, 19].

In our recent work [18] we tested the same collection of pig rectal swabs as used in the present work for the presence of porcine kobuvirus 1 (PKV-1) and RVA. The presencee of PKV-1 as a newly emerged virus, which role in gastroenteritis is still unknown, has been similar in both, healthy and diarrheic pigs, as has been observed for PAstV and PSaV in this work. However, the detection of RVA in suckling piglets, but not in weaned and fattening pigs, was significantly associated with diarrhea, confirming that this virus is a cause of gastroenteritis. In this context it is difficult to conclude, at least from our findings, that PAstV, PSaV and PKV-1 are typical enteric viruses associated with diarrhea which contrasts with conclusions by others [4, 19, 20]. This inconsistency indicates that viruses could express their pathogenicity in only a small percentage of cases as has been already hypothesized by Luo et al. [21] due to co-infection with other gastrointestinal viruses or due to immunodeficiency of animals. However, our data of the co-infection of viruses do not support this hypothesis. In two studies (this work and [18]) we did not detect strong differences between healthy and diarrheic animals with co-infection of PAstV and PSaV or in the case of rotavirus A co-infection with both PAstV and PSaV. Other studies also indicated that PAstV as a ubiquitous virus has been often found in co-infections with various viruses [7, 8]. We further speculate that the development of diarrhea in pigs is for some viruses a result of interaction the entire virome or microbiome with environment in the intestinal tract which may influence the course of viral infection. It is quite surprising that infection of gnotobiotic pigs, in which the virome is different from the virome in pigs on farms, with PSaV resulted in the development of diarrhea [22].

Despite the worldwide spread of PAst and PSa viruses in pig populations, their occurrence is significantly different in European countries, and range from approximately 20% in Germany up to 100% in Austria for PAstV [8, 23], and from 1.6% in Hungary up to 44% in Slovenia for PSaV [24]. The heterogeneous distribution of both viruses was observed also in other countries, such as USA, Canada, Korea or China [5, 16, 25, 26]. Nevertheless, in most of these studies no direct association between diarrhea and the presence of PAstV or PSaV has been observed.

In our experiments the detection of PSaV was significantly lower than PAstV, similar to findings in neighbouring countries e.g. Czech Republic or Hungary [7, 24]. In addition, PSaV together with PKV-1 and RVA [18] affected mainly suckling piglets, while in the older pigs only a few positive samples were detected in both healthy and diarrheic pigs. Some studies suggested that the piglets are protected against infection by maternal antibodies [7, 24] but our results are not in accordance with this opinion. Whether viral infections of suckling piglets could be mostly due to vertical transmission from sows will be examined in our future experiments.

The search for TGEV and PEDV has been done in diarrheic animals only, not in healthy pigs, because we believe that viral infection would be manifested with strong clinical signs (diarrhea, vomiting) which would not be observed, of course, in healthy animals. Neither virus was found in any clinical sample. However, TGEV has been already detected in neighbouring countries. TGEV is present in Hungary and causes sporadic outbreaks in pig herds [27]. Antibodies against TGEV have also been found in wild boars in Czech Republic [28]. PEDV has been detected in many European countries, including neighbouring Austria where virus was probably introduced by purchasing piglets from a German source [29].

Recently, a novel chimeric swine enteric coronavirus (SeCoV) has been identified in diseased pigs in Slovakia with clinical signs as diarrhea, vomiting and severe dehydration in young piglets resulting in a high level of mortality. Genetic analyses revealed that most of the genome of this virus was derived from TGEV, but the S-gene originated from PEDV [30]. The chimeric SeCoV detected in Slovakia in early 2015 was highly related, but not identical, to SeCoV isolates found in Italy in 2009–2012 [31] and later in Germany [32]. The isolates from Slovakia could be detected by RT-PCR with primers selected from the S gene. We also used primers selected from S gene [33] in our RT-PCR assay. Since all samples were negative for PEDV, no SeCoV isolate(s) circulated in any farms investigated in this study.

Phylogenetic analysis of the partial RdRp region of our selected PAstV positive swabs revealed a high genetic diversity among them including all known five viral lineages in pigs in Slovakia. The comparison with available sequences from GenBank database revealed that the majority of PAstV sequences belong to the lineages PAstV-2 and PAstV-4, which were also the most often detected in neighbouring countries e.g. Czech Republic and Hungary [7, 8], but also in North America [4, 21]. In addition we detected another three rare lineages PAstV-1, PAstV-3 and PAstV-5; each of them was represented by one sequence. Until now, all five lineages were detected inside Europe only in Croatia [6].

Despite the majority of PAstV sequences being farm specific, three additional different lineages - PAstV-1, PAstV-2 and PAstV-4 were found on one farm (Fig. 2, farm named RI). We did not detect more than one PAstV strain in a single pig as it was reported by Luo et al. [21]. In some sequence chromatograms, however, several smaller nucleotide peaks on the bottom of higher peaks were observed which could be the result of the presence of a minor viral isolate in the pig sample.

All 12 PSaV sequences analysed in this study fall in the phylogenetic tree into genogroup GIII, which is the most prevalent genogroup worldwide [24]. The phylogenetic tree indicated three different genotypes inside GIII (Fig. 3) as describe by Jeong et al. [34] and Dufkova et al. [7]. The RK O3 isolate showed a phylogenetic relatedness with Czech strain P38/11/CZ which was proposed as “P38/11-like” genotype [7]. This isolate was different from other isolates coming from the same farm and represented the only exception when viral isolates from the same farm did not belong to the same cluster.

Generally, most of our PSaV sequences clustered together with sequences originating from PSaV of neighbouring countries. This phenomenon indicates the circulation of related viral isolates in Central Europe. None of the Slovakian isolates has been found to be related to human sapovirus. Thus, at least at present PSaV has no zoonotic risk for human populations.

## Conclusion

This work documented the molecular detection and diversity of enteric viral agents in suckling, weaned and fattening pigs on farms in Slovakia. PAstV was found as a dominant virus species with high presence (80–99%) in investigated farms, but the presence was not depending on the health status of pigs. On the other side PSaV was found in a small percentage (around 9%) of both, healthy and diarrheic animals with higher occurrence in suckling piglets. The equal presence of both viruses in healthy and diarrheic pigs does not clearly clarify their role in gastrointestinal diseases and their detection has no diagnostic value. In addition, the phylogenetic analysis revealed all five genetic lineages of PAstV sequences. No TGEV and PEDV have been found in diarrheic pigs of different ages.

## Methods

### Farms, sample collection and preparation

The samples were collected from 17 pig farms located in Slovakia between years 2013 and 2016. The size of farms varied between 2 and 400 pigs (11 small farms) and 1.000–6.000 pigs (6 large farms). There were not the farms with all age categories of pigs because most Slovakian farms are specialized for breeding of some age categories of animals. The selection of farms was based on the information of problems with health status of pigs as inappetence, vomiting, loss of weight, growth disorders, wasting and high morbidity. Our attention was especially focused on animals with strong diarrhea.

A total of 411 rectal swabs were used in this study. The pigs belonged to three different age stages: suckling piglets (< 28 days, *n* = 143), weaned pigs (28–70 days, *n* = 147) and fattening pigs (> 70 days, *n* = 121). Rectal swabs were collected from both healthy pigs (*n* = 251) and diarrheic pigs (*n* = 160).

The swabs collected from each animal were placed into 1 ml of 0.01 mol/l PBS (Merck Milipore Corp., USA) for 30 min, and subsequently vortexed at 2000 rev. min^−1^ for 3 min and centrifuged at 12000 rpm for 5 min. After the procedure, if samples were not directly used for RNA isolation, they were stored at − 80 °C.

### RNA isolation

Total RNA was isolated from 200 μl of suspension using TRIzol Reagent (Life Technologies, USA) according to the manufacturer’s instructions, eluted in 20 μl of molecular grade water (Merck, GmbH, Germany), and used immediately or stored at − 80 °C.

### RT-PCR and nested-PCR

Reverse transcription (RT) and polymerase chain reaction (PCR) were performed separately. The synthesis of cDNA was carried out in a 20 μl reaction mixture using RevertAid Premium reverse transcriptase (ThermoScienfitic, USA) containing 5 μl of isolated RNA. The cDNA synthesis was performed according manufacturer’s instructions.

PCR was carried out in 50 μl reaction volume consisting of 1xThermoPol reaction buffer (New England Biolabs, USA), 0.2 mM dNTPs (ThermoScienfitic, USA), 0.3 μM of each primer, 0.5 U Taq DNA polymerase (New England Biolabs, USA), 4 μl cDNA and molecular grade water. Primers used for PCR detection of TGEV, PEDV, PSaV and PAstV are shown in Table 1. A single PCR was carried out for the detection of TGEV, PEDV and PSaV. A semi-nested PCR was used for PAstV detection. PCR primers panAV-F11 (forward), panAV-F12 (forward) and panAV-R1 (reverse) were used in an initial RT-PCR, while primers panAV-F21 (forward), panAV-F22 (forward) and panAV-R1 were used in the semi-nested PCR (Table 1).

**Table 1:** Sequence of primers used for RT-PCR and sequencing

Virus	Primer	Sequence (5′-3′)	Region	Size (bp)	References
*PAstV*	panAV-F11	GARTTYGATTGGRCKCGKTAYGA	RdRp	420–400	[36]
	panAV-F12	GARTTYGATTGGRCKAGGTAYGA			
	panAV-F21	CGKTAYGATGGKACKATHCC			
	panAV-F22	AGGTAYGATGGKACKATHCC			
	panAV-R1	GGYTTKACCCACATICCRAA			
*PSaV*	PECVcapsidF	CTCATCAACCCTTTTGAAAC	Capsid protein	757	[26]
	PECVcapsidR	AAAGCATGATGTTGTTAGGC			
*TGEV*	T1	GTGGTTTTGGTYRTAAATGC	Spike protein	858	[33]
	T2	CACTAACCAACGTGGARCTA			
*PEDV*	P1	TTCTGAGTCACGAACAGCCA	Spike protein	650	
	P2	CATATGCAGCCTGCTCTGAA			

The PCR program used for detection of all investigated viruses had the following thermal profile: initial denaturation at 95 °C for 1 min, followed by 35–37 cycles with denaturation at 95 °C for 30 s, annealing at 50–53 °C for 1 min, extension at 68 °C for 1 min and final extension at 68 °C for 5 min.

The size of PCR products was analyzed by 2% agarose gel electrophoresis and visualized by Gel Doc EZ imager (Bio-Rad Laboratories, USA) after staining with GelRed™ (Biotum, USA).

While tests for PAstV and PSaV were done on samples collected from healthy and diarrheic pigs (*n* = 411), tests for TGEV and PEDV were done only on samples from diarrheic samples (*n* = 160).

### Sequencing of DNA and phylogenetic analysis

PCR amplicons (*n* = 25) from PAstV positive samples from 13 farms and PCR amplicons (*n* = 12) obtained from PSaV positive samples originating from 6 farms were used for sequencing and phylogenetic analysis. The selection of PCR amplicons was carried out on the basis of their quality and to cover healthy and diarrheic pigs from the entire geographic area. The purification of PCR amplicons and sequencing in both directions using Sanger’s method employing fluorescently labelled ddNTPs was carried out by the commercial company Microsynth Austria GmbH (Austria). The chromatograms were checked and edited by the computer programme SeqMan in our laboratory. The nucleotide sequences were deposited into GenBank under accession numbers MG051040-MG051076.

The nucleotide sequences were aligned by the MegAlign program (Lasergene, DNASTAR, Inc., USA). The phylogenetic trees were constructed by the neighbour-joining method using the Kimura-2 parameter using program MEGA6 [35].

### Statistical analysis

Statistical analyses of data were performed by chi-square (χ^2^) test with confidence limits of 95%, *P* < 0.05 or 99%, *P* < 0.01 using GraphPad Prism 5 for Windows (GraphPad Software, USA).

## Abbreviations

PAstV: Porcine astrovirus
PEDV: Porcine epidemic diarrhea virus
PKV-1: Porcine kobuvirus 1
PSaV: Porcine sapovirus
RdRp: RNA-dependent RNA polymerase
TGEV: Transmissible gastroenteritis virus
